# A Golgi-based KDELR-dependent signalling pathway controls extracellular matrix degradation

**DOI:** 10.18632/oncotarget.3270

**Published:** 2014-12-18

**Authors:** Carmen Ruggiero, Giorgia Fragassi, Mauro Grossi, Benedetta Picciani, Rosaria Di Martino, Mirco Capitani, Roberto Buccione, Alberto Luini, Michele Sallese

**Affiliations:** ^1^ Unit of Genomic Approaches to Membrane Traffic, Fondazione Mario Negri Sud, Santa Maria Imbaro, Chieti, Italy; ^2^ Laboratory of Tumour Cell Invasion, Fondazione Mario Negri Sud, Santa Maria Imbaro, Chieti, Italy; ^3^ Institute of Protein Biochemistry National Research Council, Naples, Italy; ^4^ Current address: Institut de Pharmacologie Moléculaire et Cellulaire CNRS and Associated International Laboratory (LIA) NEOGENEX CNRS and University of Nice-Sophia-Antipolis, Valbonne, France

**Keywords:** Cancer cell invasion, cell signalling, Src family kinases

## Abstract

We recently identified an endomembrane-based signalling cascade that is activated by the KDEL receptor (KDELR) on the Golgi complex. At the Golgi, the KDELR acts as a traffic sensor (presumably via binding to chaperones that leave the ER) and triggers signalling pathways that balance membrane fluxes between ER and Golgi. One such pathway relies on Gq and Src. Here, we examine if KDELR might control other cellular modules through this pathway. Given the central role of Src in extracellular matrix (ECM) degradation, we investigated the impact of the KDELR-Src pathway on the ability of cancer cells to degrade the ECM. We find that activation of the KDELR controls ECM degradation by increasing the number of the degradative structures known as invadopodia. The KDELR induces Src activation at the invadopodia and leads to phosphorylation of the Src substrates cortactin and ASAP1, which are required for basal and KDELR-stimulated ECM degradation. This study furthers our understanding of the regulatory circuitry underlying invadopodia-dependent ECM degradation, a key phase in metastases formation and invasive growth.

## INTRODUCTION

Eukaryotic cells can be thought of as assemblies of functional modules operating through specialized physical structures, or organelles, that support a variety of processes such as metabolism, membrane transport, cell division, autophagy, and apoptosis [1-3]. These modules are partially independent of each other, but their activities must be coordinated to achieve harmonic cellular behaviours [2, 3].

In recent years, we have begun to analyse the mechanisms by which the secretory pathway, one of the main cellular functional modules, maintains its homeostasis and coordinates its activity with those of other modules. Membrane traffic is a fundamental process by which a third of the mammalian proteins are transported from their site of synthesis, the endoplasmic reticulum (ER), through a series of anatomically separated membranous compartments until they reach their cellular destinations in correctly processed forms. We have shown that membrane fluxes leaving the ER for the Golgi result in the activation of a traffic sensor, called KDEL receptor (KDELR) at the cis-Golgi [4-6]. The KDELR is a seven-transmembrane domain protein belonging to the PQ-loop protein family [7] that is distantly related to the G protein-coupled receptor (GPCR) superfamily [8, 9], resembles the GPCRs in topology and fold of the transmembrane helices [8, 9]. Strong though circumstantial evidence indicates that the activation of the KDELR by transport is mediated by the chaperones, a family of ER resident proteins that carry a KDEL signal at their C terminus and cycle between the ER and the Golgi [4-6] (of note, the KDELR has been discovered and characterized as a mediator of the recycling to the ER of chaperones leaked out from this organelle during traffic [10-12]). When membrane export from the ER brings chaperones to the Golgi, these proteins bind the KDELR [13], which then activates a Golgi pool of the heterotrimeric G proteins G_q_ and G_s_. G_s_ activates a signalling cascade at the cis-Golgi resulting in the activation of retrograde membrane transport [4], while G_q_, acts by inducing the activation of Src (for brevity, from here onward, we will refer to the Src family proteins as Src), which phosphorylates a number of proteins, and activates anterograde traffic. Thus, a device based on the KDELR and two signalling pathways appears to sense incoming traffic into the Golgi and activate antero- and retro-grade transport to counteract the accumulation of membrane/proteins that would result from a non-compensated continuous arrival of traffic to the Golgi from the ER [14-16], helping to maintain Golgi homeostasis. In addition, the activation of the KDELR causes the phosphorylation of proteins involved in functions other than traffic, and also enhances the expression of genes that belong to functional modules such as mitochondrial and energy metabolism, lipid metabolism, protein degradation, autophagy and motility [4]. These responses indicate the presence of mechanisms that coordinate membrane transport with other cellular functions.

In this study, we begin to examine the molecular basis of the coordination between transport and other modules, starting from the role of Src and the consequent tyrosine phosphorylation cascade in the Golgi response to traffic. As indicated by our early observations, while active Src appears at the Golgi early during arrival of membrane transport, it later spreads to more peripheral cellular locations [6]. Src is the best characterized member of the Src family kinases, that includes nine members [17, 18] and regulates a variety of functions, including cell proliferation, differentiation, survival, adhesion, migration and matrix invasion [17]. Here, prompted by the established role of Src in the formation and function of invadopodia, and hence in matrix invasion, we have examined whether the traffic- and KDELR-initiated Src signalling pathway might be involved in invadopodia-dependent ECM degradation.

In cancer cells, invadopodia mediate the degradation of the ECM via matrix metalloproteinase (MMP) activity [19]. Invadopodia formation requires the engagement of cell-surface integrins by the ECM and tyrosine-kinase-based signalling [20, 21]. Growth factors can also trigger invadopodia formation/activity [22-27] through the phosphorylation and/or activation of invadopodia proteins, including Src. Overexpression/activation of Src is sufficient to elicit invadopodia formation and ECM degradation in fibroblasts (which otherwise lack invadopodia) and breast carcinoma cells [28, 29], while treatment with Src inhibitors or expression of kinase-inactive Src mutants, inhibits invadopodia formation and ECM degradation [29, 30]. Src regulates the activity of key invadopodia proteins either indirectly or by direct phosphorylation as shown for Arf-GTPase-activating protein (GAP) ASAP1 [29, 31, 32] and cortactin [33, 34].

The importance of invadopodia in the process of cancer metastasis formation is supported by studies that have reported enrichment of invadopodia markers (e.g. cortactin and Tks5/FISH) at the invading front of human tumour cells [35, 36]. Furthermore, in different model systems, invadopodia formation has been shown to be associated with cancer invasion, including tumour metastasis in nude mice [37, 38]. Finally, intravital imaging studies have shown that carcinoma cells in the process of intravasation extend invadopodia-like protrusions that can penetrate blood vessel walls [39, 40].

To examine the role of the KDELR-Src signalling cascade in ECM degradation, we have examined the effect of activating this pathway on the degradation of matrix by invasive metastatic human cell lines [41, 42]. We find that the activation of the KDELR increased the degradation activity of A375MM cells, while inhibition of KDELR signalling inhibited the cell-degrading activity. The enhanced degradation promoted by the KDELR involved the activation of Src and the phosphorylation of cortactin and ASAP1 at the invadopodia. The effects of the KDELR on matrix degradation were comparable to those of serum, the most effective degradation inducer known so far. Thus, a protein cycling in a transport-dependent fashion across early stations of the secretory pathway controls the ability of the cell to interact with its extracellular matrix through endomembrane based signalling.

## RESULTS

As noted, Src is absolutely required for invadopodia formation and ECM degradation [28-30]. In the following, we examine: a) the effect of activators of the KDELR on matrix degradation, b) the role of the KDELR in the activation of Src at sites of matrix degradation, or invadopodia and, c) the role of the KDELR-induced phosphorylation of Src substrates such as cortactin and ASAP1 in matrix degradation.

### Activation of the KDELR promotes degradation of the ECM in a human melanoma cell line

**Figure 1:**
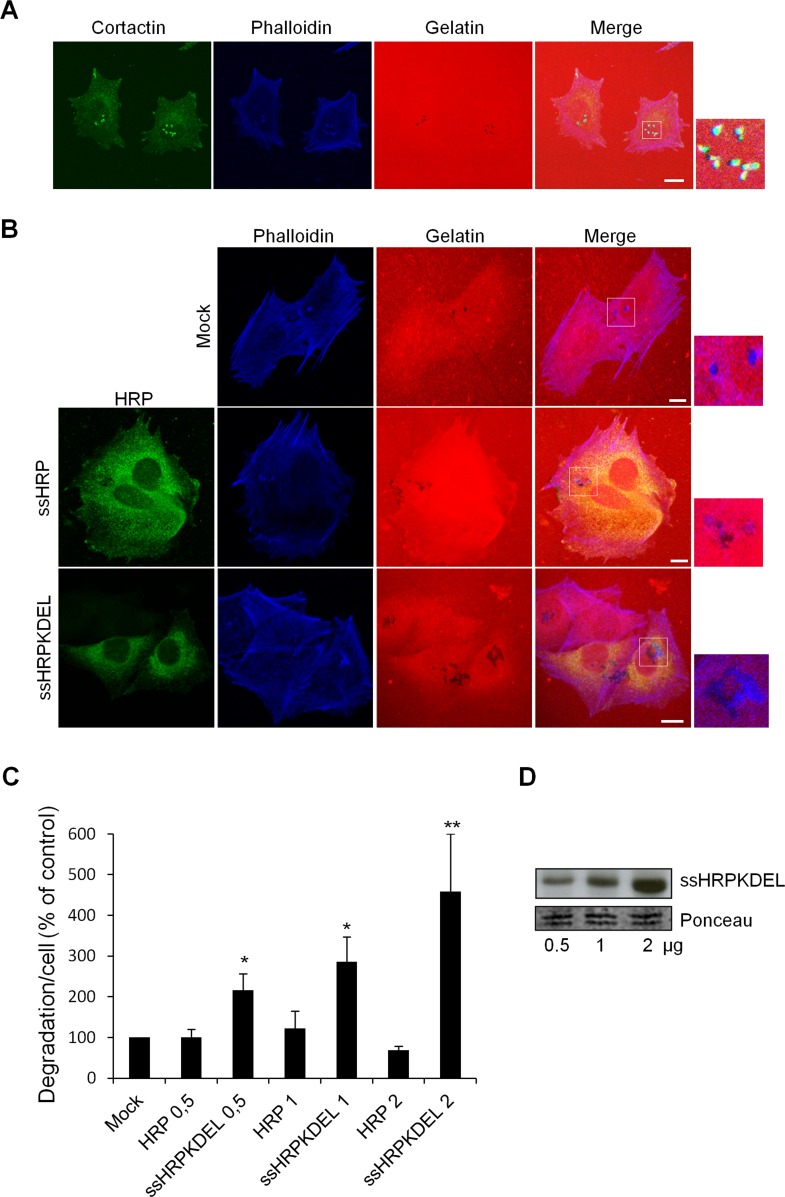
KDELR stimulation by overexpression of the KDELR ligand ssHRP^KDEL^ increases degradation of the ECM (A) A375MM cells were grown in complete medium (with 10% serum) on rhodamine-conjugated crosslinked gelatine (red) for 16 h in the presence of BB94. Following BB94 wash-out, the cells were incubated for a further 3 h, and then fixed and stained with phalloidin (blue) and cortactin (green). Merged images of blue green and red signals are also shown (Merge). Invadopodia are evident in the enlargement of the boxed region (small right panel: green, blue and red signals). (B) A375MM cells were transfected with empty vector (Mock), ssHRP (2 μg), or ssHRP-KDEL (2 μg), and treated as in A. The cells were then fixed and stained with an anti-HRP antibody (green) and phalloidin (blue). Merged images of red and blue(Mock) and red, blue and green signals (ssHRP and ssHRP ssHRP^KDEL^) are also shown (Merge). Invadopodia are shown in the enlargements of the boxed regions (small right panels: blue and red signals). (A, B). Scale bars, 10 μm. The images are representative of three independent experiments. (C) Cells transfected with increasing concentrations (0.5, 1, 2 μg) ssHRP or ssHRP^KDEL^ were processed as in A. Data are degradation area per cell (% of control), as means ±SEM from three independent experiments, with at least 100 cells quantified per experiment. *p<0.05, **p <0.01, compared to Mock (control) cells (t-test). (D) A375MM cells transfected with increasing concentrations (0.5, 1, 2 μg) of ssHRP^KDEL^ were lysed and analyzed by immunoblotting with an anti-HRP antibody (upper panel). A region of the nitrocellulose membrane stained by ponceau red is shown as a loading control (lower panel).

A375MM cells are a metastatic variant of an established human melanoma cell line [43]. A375MM cells plated on a cross-linked fluorophore-conjugated gelatine matrix form degradation areas co-localizing with invadopodia markers, including actin, cortactin, dynamin-2 and phosphorylated tyrosines [20, 42]. Fig. 1A shows these dark patches in the fluorescent gelatine matrix overlapped by cortactin and actin dots (invadopodia).

To determine whether activation of the KDELR can induce matrix degradation, we activated the KDELR by transfecting the cells with the soluble-secreted variant of horseradish peroxidase bearing the KDEL motif at its C-terminus (ssHRP^KDEL^) [6]. This artificial KDELR ligand is expressed in the lumen of the ER and then transported to the Golgi, where it binds to the KDELR through its KDEL sequence; this leads to persistent stimulation of the KDELR [6]. A375MM cells transfected with increasing amounts of ssHRP^KDEL^ (0.5, 1.0, 2.0 μg) were plated on fluorophore-conjugated gelatine-coated coverslips in the presence of BB94 (metalloproteinases inhibitor widely used to synchronize ECM degradation [42]), and left overnight. The day after, BB94 was washed-out and 3 hours later the cells were fixed and stained with an anti-HRP antibody, for the identification of the transfected/stimulated cells and phalloidin to identify the invadopodia (Fig. 1B). In this study, unless differently specified, we considered as mature invadopodia the actin rich structures (identified by phalloidin staining) that overlap dark patches of degradation. The degradation areas were evaluated as previously described [42] and were expressed as the mean degradation area per cell.

Chronic stimulation of the KDELR by overexpression of ssHRP^KDEL^ increased ECM degradation three-fold, with this activity correlating with the level of transfected ssHRP^KDEL^ (Fig. 1B, C). The correlation between the degradation response and the expression levels of ssHRP^KDEL^ was validated by Western blot analysis (Fig. 1D). The increased area of degradation also correlated with a higher number of degradation patches and of mature invadopodia in ssHRP^KDEL^ transfected cells compared to controls. Specifically, controls showed an average of 2,3±1 mature invadopodia/cell while ssHRP^KDEL^ showed an average of 6±1 mature invadopodia/cell. In addition, the controls showed an average of 3.1±1 degradation patches/cell while ssHRP^KDEL^ showed an average of 8±1.5 degradation patches/cell. Finally, the average size of each degradation patch was about 0.7±0.2 μm in control cells while it was about 1±0.2 μm in ssHRP^KDEL^ transfected cells. As a control, cells were transfected with empty vector (mock), and with increasing amounts (0.5, 1.0, 2.0 μg) of the soluble secreted variant of horseradish peroxidase lacking the KDEL motif (ssHRP). Neither the empty vector nor ssHRP affected the ECM degradation activity (Fig. 1B, C).

**Figure 2:**
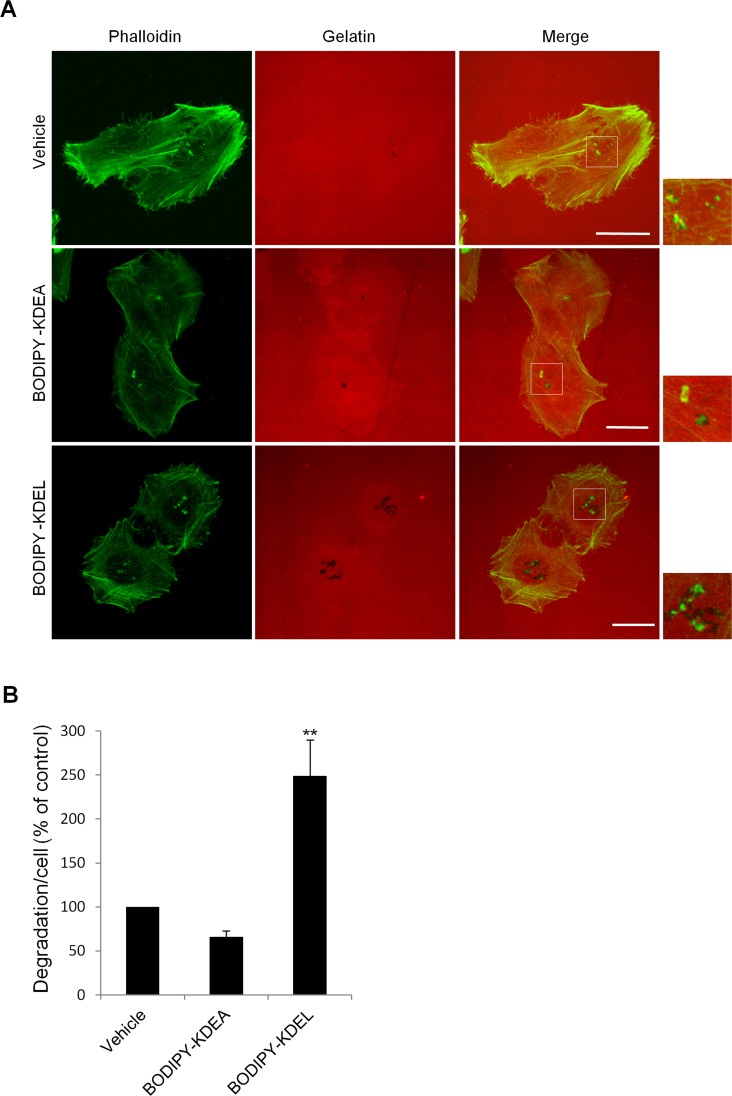
KDELR stimulation by Bodipy-KDEL promotes ECM degradation (A) A375MM cells were grown on rhodamine-conjugated crosslinked gelatine (red) for 16 h in the presence of BB94. Following BB94 wash-out, the cells were incubated for a further 3 h with the membrane permeant KDELR agonist Bodipy-KDEL (3 μM) or the control peptide Bodipy-KDEA (3 μM) (as indicated). As an additional control, the cells were incubated with vehicle alone (Vehicle). After fixing, the cells were stained with phalloidin (green). Merged images of red and green signals are also shown (Merge). Invadopodia are shown in the enlargements of the boxed regions (small right panels: green and red signals). Scale bars, 10 μm. The images are representative of three independent experiments. (B) Quantification of the degradation area per cell. Data are degradation area per cell (% of control), as means ±SEM of three independent experiments, with at least 100 cells quantified per experiment. ** p<0.01, compared to Vehicle cells (t-test).

We also investigated the effects of activating the KDELR acutely on matrix degradation stimulating the KDELR by a cell-permeable ligand. For this, we exploited an artificial ligand constituted by the KDEL tetrapeptide conjugated to Bodipy; this construct can cross biological membranes and activate the KDELR, as characterized in our recent study [5]. A375MM cells were plated on gelatine in the presence of BB94, and then the protease inhibitor was washed-out, and the cells were treated for 3 h with Bodipy-KDEL. As a control, cells were incubated with the vehicle alone or with Bodipy-KDEA, a peptide motif that does not bind to or activate the KDELR [5, 44]. The degradation area of Bodipy-KDEL–treated cells was twice that of control cells (Fig. 2A, B). In the Bodipy-KDEA–treated cells, ECM degradation was comparable to that in vehicle-treated cells. Further, control cells showed an average of 2.4±0.3 degradation patches/cell while Bodipy-KDEL–treated cells showed an average of 4.9±0.3 degradation patches/cell. The average size of each degradation patch was about 0.8±0.1 μm in control cells while it was about 1.1±0.1 μm in Bodipy-KDEL–treated cells. Thus, in KDELR-stimulated cells (in both chronic and acute fashions), the increased mean degradation area per cell was the result of a higher number of degradation areas and of larger degradation patches.

In order to investigate if KDELR activation might affect the development and maturation of invadopodia, we treated cells with Bodipy-KDEL and quantified the number of cytoskeletal dots labelled with both phalloidin and cortactin but lacking matrix degrading activity (immature invadopodia) and the number of similar structures associated with matrix degradation (mature invadopodia). The Bodipy-KDEL treatment increased the number of mature invadopodia while the number of immature invadopodia was only slightly affected by KDELR stimulation (Suppl. Fig. 1).

### KDELR1 and KDELR2 control ECM degradation

The human genome contains three highly homologous KDELR genes, known as KDELR1, KDELR2 and KDELR3 [45] whose functional specificity is still unknown. We investigated the involvement of each of these KDELRs in ECM degradation.

**Figure 3:**
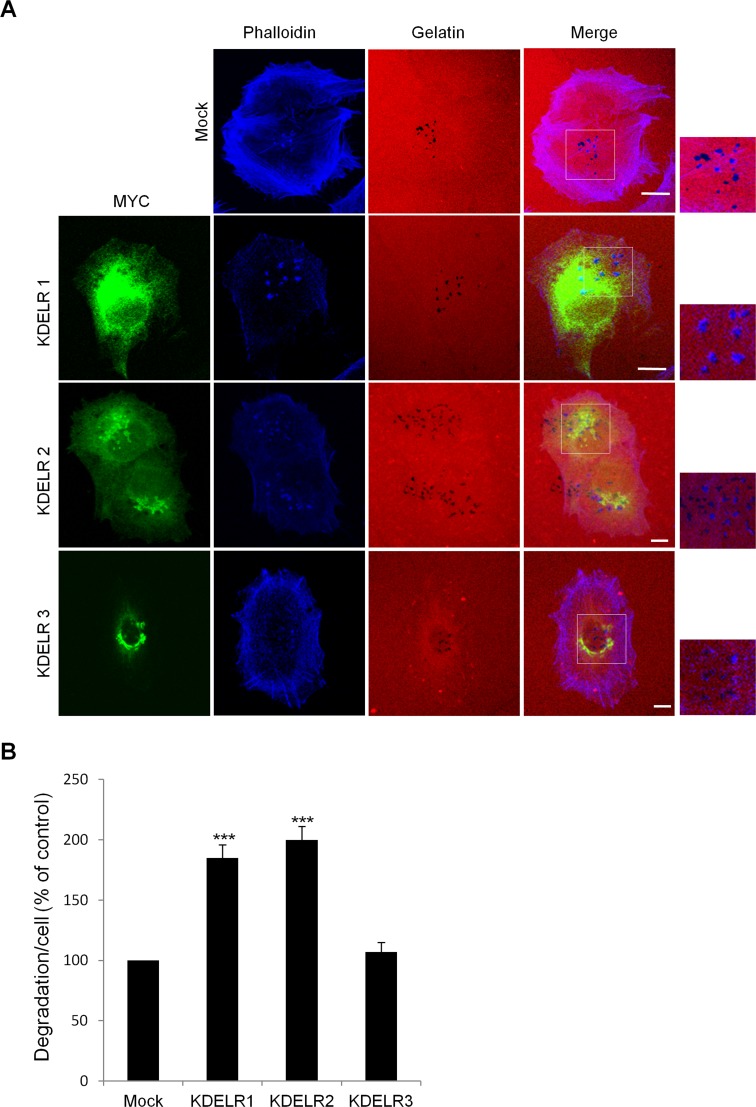
The ECM degradation process is differentially regulated by the KDELR isoforms (A) A375MM cells were transfected with empty vector (Mock) or the myc-tagged KDELR isoforms, for KDELR1, KDELR2 and KDELR3, and grown on rhodamine-conjugated (red) crosslinked gelatine for 16 h in the presence of BB94. Following BB94 wash-out, the cells were incubated for a further 3 h, then fixed and stained with an anti-myc antibody to visualise transfected cells (green) and phalloidin (blue). Merged images of red and blue (Mock) and red, blue and green signals (KDELRs) are also shown (Merge) Invadopodia are shown in the enlargements of the boxed regions (small right panels: blue and red signals). Scale bars, 10 μm. The images are representative of three independent experiments. (B) Quantification of the degradation area per cell. Data are degradation area per cell (% of control), as means ±SEM from three independent experiments, with at least 100 cells quantified per experiment. ***p <0.001, compared to Mock cells (t-test).

For this, we took advantage of specific features of the KDELR, which autoactivates when overexpressed [6]. This has been monitored via the redistribution of activated KDELR2 from the Golgi to the ER (Suppl. Fig. 2; [6]). A375MM cells were transfected with myc-tagged KDELR2, plated on gelatine-coated coverslips, and assayed for ECM degradation. In these cells, the extent of degradation was double that compared to mock-transfected cells (Fig. 3A, B). Considering the high degree of homology between the three KDELR isoforms, we hypothesized that when overexpressed at high levels, KDELR1 and KDELR3 would also self-activate and redistribute to the ER, similar to KDELR2. Upon expression of high levels of KDELR1 and KDELR3, these redistributed to the ER, indicating that in analogy to KDELR2, overexpressed KDELR1 and KDELR3 can also self-activate (Suppl. Fig. 2). A375MM cells were transfected with the myc-tagged KDELR1 and KDELR3, plated and assayed for ECM degradation. In KDELR1-transfected cells, the extent of degradation was almost double that compared to mock-transfected cells (Fig. 3A, B). Instead, the degradation area of cells transfected with KDELR3 was only slightly increased (Fig. 3A, B). Besides increasing the ECM degradation area, KDELR1 and KDELR2 overexpression increased the number of degradation patches and mature invadopodia. Specifically, controls showed an average of 2.9±0.1 degradation patches/cell while KDELRs overexpressing cells showed an average of 4.3±0.2 degradation patches/cell. Mature invadopodia/cells increased from 2.3±0.1 in control to 3.5±0.2 in KDELRs-overexpressing cells. Finally, the average size of each degradation patch was about 0.6±0.1 μm in control cells while it was about 0.9±0.1 μm in KDELR transfected cells.

These results indicate that the activation of KDELR1 and KDELR2 enhances degradation of the ECM by regulating invadopodia formation.

To determine the intensity of the KDELR-dependent ECM degradation, we compared the levels of degradation obtained by raising the serum concentration (serum growth factors are the most potent known regulators of ECM degradation), with those obtained by KDELR activation. The A375MM cell ECM-degradation assay was carried out in the presence of serum concentrations ranging from 0% to 50%. As expected, without serum, there was no degradation activity, while the degradation started at 1% serum and reached a plateau at 30% to 50% serum (Suppl. Fig. 3). The maximal degradation was two-fold that observed in standard growth medium (with 10% serum), the condition typically used in ECM degradation assays. Remarkably, ECM degradation triggered by KDELR activation in standard growth medium (with 10% serum) was comparable to that which was maximally obtainable at high serum concentrations (compare Suppl. Fig. 3). Notably, KDELR2-overexpressing cells increased ECM degradation significantly even in the presence of up to 50% serum concentrations (Suppl. Fig. 3).

### The inhibition of the KDELR reduces ECM degradation

**Figure 4:**
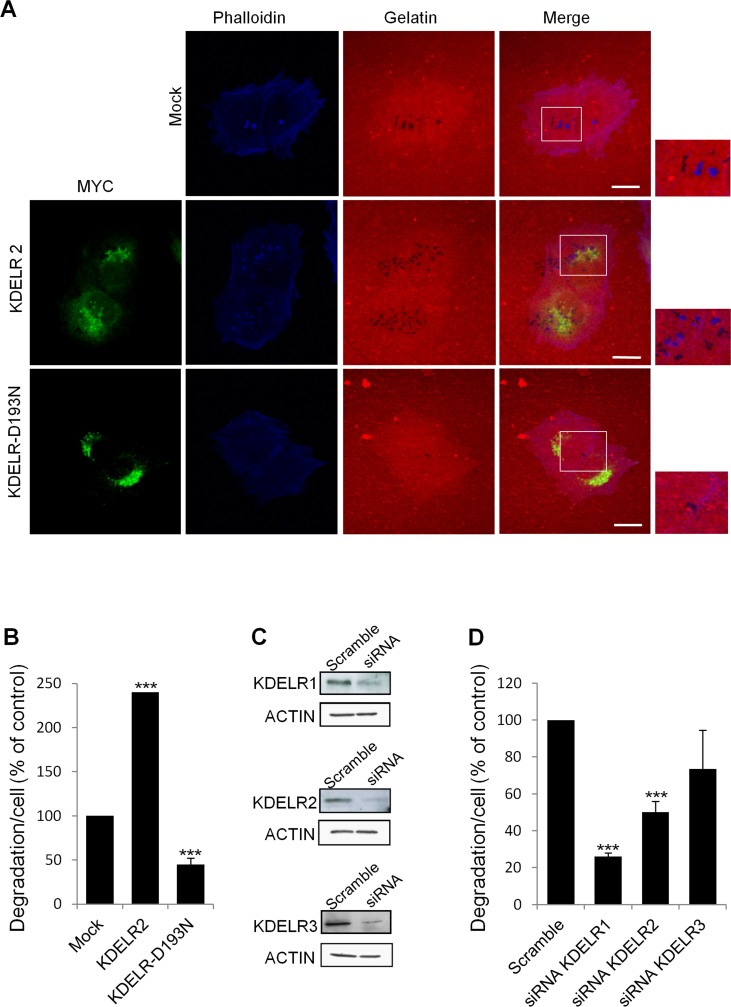
KDELR inhibition impairs degradation of the ECM (A) A375MM cells were transfected with empty vector (Mock), KDELR2-myc or KDELR-D193N-myc (as indicated) for 24 h, and grown on rhodamine-conjugated crosslinked gelatine (red) for 16 h in the presence of BB94. Following BB94 wash-out, the cells were incubated for a further 3 h and then fixed and stained with -phalloidin (blue, Mock) and an anti-myc antibody (green, KDELR2-myc and KDELR-D193N-myc). Merged images of red and blue (Mock) and red, blue and green signals (KDER2 and KDELR-D193NL) are also shown. Invadopodia are shown in the enlargements of the boxed regions (small right panels: blue and red signals). Scale bars, 10 μm. The images are representative of at least four independent experiments. (B) Quantification of the degradation area per cell. Data are degradation area per cell (% of control), as means ±SEM of four independent experiments, with at least 100 cells quantified per experiment. ***p <0.001, compared to Mock cells (t-test). (C) A375MM cells were treated without (Scramble) or with siRNAs targeting KDELR1, KDELR2 and KDELR3 (siRNA KDELR) for 96 h. KDELR1, KDELR2, and KDELR3 expression levels were assessed by Western blotting, using a pan anti-KDELR antibody, an antibodies specifically directed against KDELR2 and KDELR3, respectively. Actin was used as loading control. (D) A375MM cells were treated as in C, and then 72-h post interference they were plated for 24 h on rhodamine-conjugated gelatine in the presence of BB94. Following the BB94wash-out, the cells were incubated for a further 3 h, then fixed and scored for their ability to degrade the ECM. Data are degradation area per cell (% of control), as means ±SEM from three independent experiments, with at least 100 cells quantified per experiment. ***p <0.001, compared to Scrambled cells (t-test).

As a complementary approach to demonstrate the involvement of the KDELR in ECM degradation, we impaired KDELR functions by multiple methods. First, the A375MM cells were transfected with the myc-tagged KDELR-D193N dominant-negative mutant [5, 6], and subjected to the ECM degradation assay in standard growth medium. Overexpression of the dominant-negative receptor resulted in 50% inhibition of ECM degradation, as compared to mock-transfected cells (Fig. 4A, B). Second we knocked-down the KDELRs individually and evaluated the ECM degradation and invadopodia formation. The A375MM cells were transfected with a SMARTpool of siRNAs targeting KDELR1, KDELR2 or KDELR3, and 72 h later they were analysed for ECM degradation. The silencing of KDELR1 and KDELR2 strongly decreased the ECM degradation as compared to the cells transfected with the scrambled siRNA (Fig. 4C, D). The silencing of KDELR3 caused a minor inhibition of ECM degradation (Fig. 4C, D), as compared to control cells. The KDELR2 and KDELR3 depletion was efficient, as assessed by Western blotting using isoform-specific antibodies and quantitative real time PCR (qPCR) (Fig. 4C and Suppl. Fig. 4). KDELR1 was evaluated using a pan-antibody (Fig. 4C) as KDELR1-selective antibodies were not available and qPCR (Suppl. Fig. 4). Overall, these data confirm the specific involvement of KDELRs in the control of ECM degradation.

When the KDELR1 and KDELR2 functions were impaired by siRNAs, we also observed a reduced number of invadopodia, degradation patches/cell and a slight reduction of mean size of the degradation patches. Specifically, the number of mature invadopodia decreased from 2.5±0.1 in the control to 0.8±0.3 per cell in cells treated with anti-KDELR siRNAs, and the number of degradation patches/cell decreased from 3.5±0.4 in control to 1.5±0.3 in cells transfected with KDELRs siRNA. Finally, the average size of each degradation patch was about 1±0.3 μm in control cells while it was about 0.7±0.2 μm in siRNA-interfered cells. Similar data were obtained using two independent siRNAs for KDELR1 and KDELR2 (Suppl. Fig. 5).

### KDELRs modulate ECM degradation also in MDA-MB-231 cells

**Figure 5:**
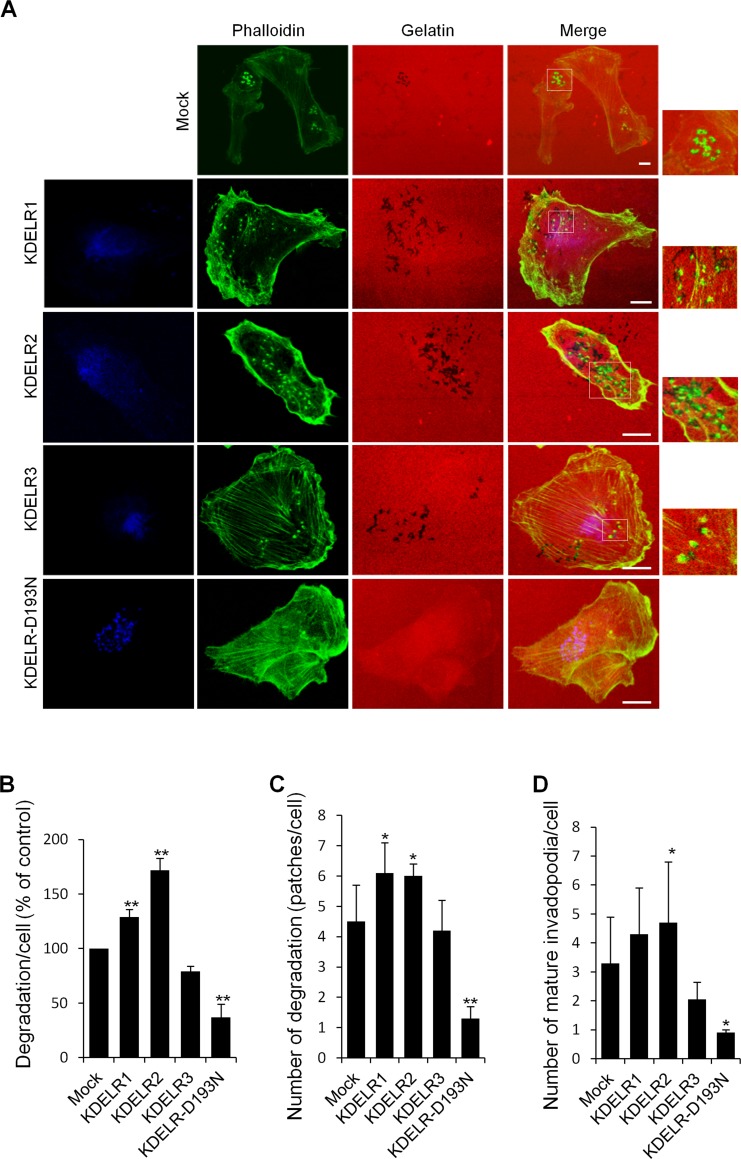
KDELR modulates the degradation of the ECM and the formation of invadopodia in MDA-MB-231 cells (A) MDA-MB-231 cells were transfected with empty vector (Mock) or the myc-tagged KDELR isoforms, for KDELR1, KDELR2, KDELR3, or KDELR-D193N-myc (as indicated) and grown on rhodamine-conjugated (red) crosslinked gelatine for 16 h in the presence of BB94. Following BB94 wash-out, the cells were incubated for 90 min and then fixed and stained with phalloidin (green) and an anti-myc antibody (blue, KDELR2-myc and KDELR-D193N-myc). Merged images of red and green (Mock) and red, green and blue signals (KDELRs and KDELR-D193N) are also shown (Merge) Invadopodia are shown in the enlargements of the boxed regions (small right panels: green and red signals). Scale bars, 10 μm. The images are representative of three independent experiments. (B) Quantification of the degradation area per cell (% of control). (C) Quantification of the number of degradation patches per cell (D) Quantification of the number of mature invadopodia per cell. Data are expressed, as means ±SEM of three independent experiments, with at least 50 cells quantified per experiment. **p <0.01, **p <0.05 compared to Mock cells (t-test).

In order to understand if the KDELR-dependent modulation of ECM degradation observed in A375MM melanoma cells is a peculiarity of this cell line or represents a general regulatory mechanism, we investigated ECM degradation in MDA-MB-231 cells, an invasive human breast carcinoma cell line, commonly used to study ECM degradation [29]. MDA-MB-231 cells were transfected with myc-tagged KDELRs, plated on gelatine-coated coverslips, and assayed for ECM degradation. Also in these cells, the extent of degradation, significantly increased over those in mock-transfected cells (Fig. 5). In MDA-MB-231 cells the quantification of the number of degradation patches and the number of invadopodia are tricky because KDELR activation causes the enlargement of each degradation patch that form pooled degradation areas (Fig. 5).

Furthermore, MDA-MB-231 cells were transfected with a myc-tagged KDELR-D193N dominant-negative mutant [5, 6] and subjected the cells to the ECM degradation assay in standard growth medium. Overexpression of the dominant-negative receptor resulted in 70% inhibition of ECM degradation, as compared to the mock-transfected cells (Fig. 5). The number of degradation patches/cell and invadopodia/cell decreased as well (Fig. 5). These data indicate that the KDELR controls the ECM degradation process also in MDA-MB-231 cells, supporting a general role of the KDELR regulatory circuit.

### KDELR activity modulates the levels of active Src at invadopodia

Since, as noted, Src is required for invadopodia formation and ECM degradation [28-30], we examined whether the KDELR might activate Src at the invadopodia and hence control ECM degradation via Src. First, A375MM cells were transfected with ssHRP^KDEL^, subjected to the ECM degradation assay, and stained with an *anti*-*p*Src *antibody*.

**Figure 6:**
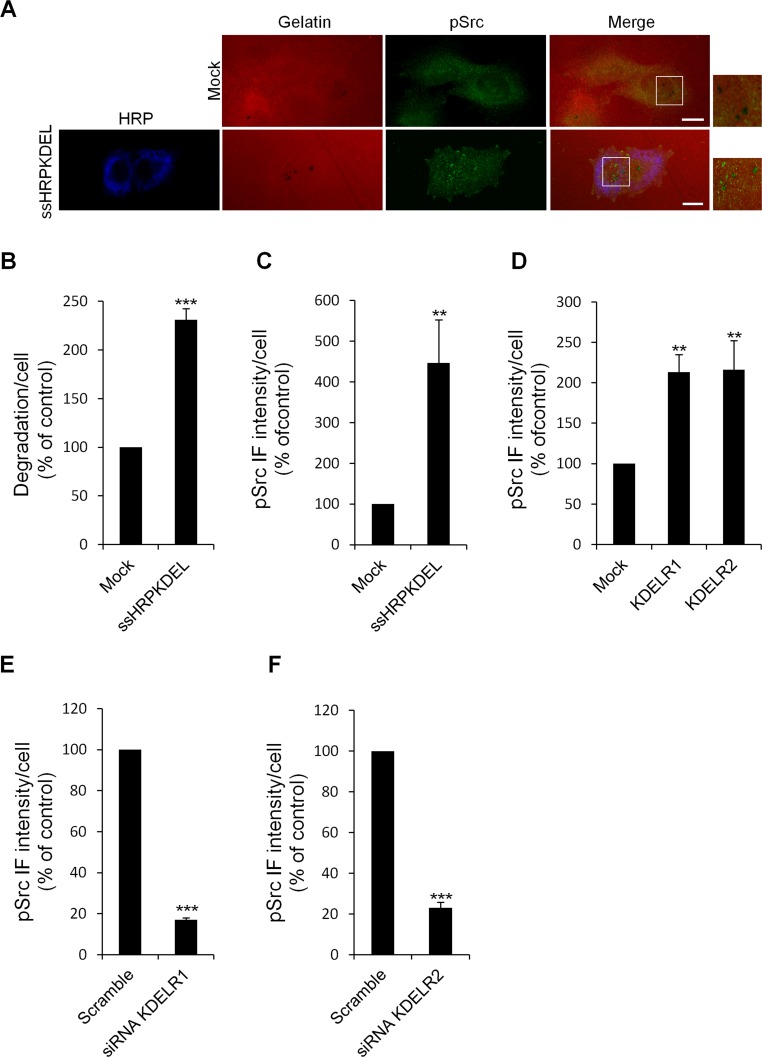
KDELR stimulation increases the levels of active Src in the cell regions overlapping the ECM degradation patches (A) A375MM cells were transfected with the empty vector (Mock) or ssHRP^KDEL^ and grown on rhodamine-conjugated crosslinked gelatine (red) for 16 h in the presence of BB94. Following BB94 wash-out, the cells were incubated for a further 3 h, and then fixed and stained for pSrc (pTyr 419, green). An anti-HRP antibody (blue) was used to visualise ssHRP^KDEL^-transfected cells. Merged images of red and green (Mock) and red, green and blue signals (ssHRP^KDEL^) are also shown (Merge). pSrc immunofluorescence overlapping the degradation patches are shown in the enlargements of the boxed regions (small right panels: red and green signals). Scale bars, 10 μm. The images are representative of three independent experiments. (B) Quantification of the degradation area per cell. Data are degradation area per cell (% of control), as means ±SEM of three independent experiments, with at least 100 cells quantified per experiment. (C) Quantification of pSrc immunofluorescence levels in the cell regions overlapping the ECM degradation patches. Data are means ±SEM of pSrc immunofluorescence per cell (% of control), from three independent experiments, with at least 100 cells quantified per experiment.. (D) A375MM cells were transfected with the empty vector (Mock) or the myc-tagged KDELR isoforms, KDELR1 and KDELR2, and grown on rhodamine-conjugated crosslinked gelatine for 16 h in the presence of BB94. Following BB94 wash-out, the cells were incubated for a further 3 h, and then fixed and labelled for pSrc. Data are means ±SEM, as indicated for C. (E-F) A375MM cells were treated without (Scramble) or with siRNA targeting KDELR1 or KDELR2 (siRNA KDELR) for 96 h. Seventy-two hours post interference, the cells were plated for 24 h on rhodamine-conjugated gelatine in the presence of BB94. Following the wash-out of the BB94, the cells were incubated for a further 3 h, then fixed and labelled for pSrc. Data are means ±SEM, as indicated for C. (B-E) ***p <0.001, **p <0.01 compared to Mock cells (t-test).

In these cells, the levels of active Src in the cell regions overlapping the ECM degradation patches were significantly increased (by four-fold as compared to those transfected with empty vector) (Fig. 6A, C). As expected, these cells showed increased ECM degradation (Fig. 6A, B).

The pSrc levels were also measured in KDELR-transfected cells. To this end, A375MM cells were transfected with KDELR1 and KDELR2 isoforms individually, and then subjected to the ECM degradation assay. In both KDELR1-overexpressing and KDELR2-overexpressing cells the increased degradation correlated with increased pSrc levels in the cell regions overlapping the ECM degradation patches (Fig. 6D). KDELR might thus be involved in local control of the ECM degradation process, through regulation of Src activity at the sites where matrix degradation occurs.

**Figure 7:**
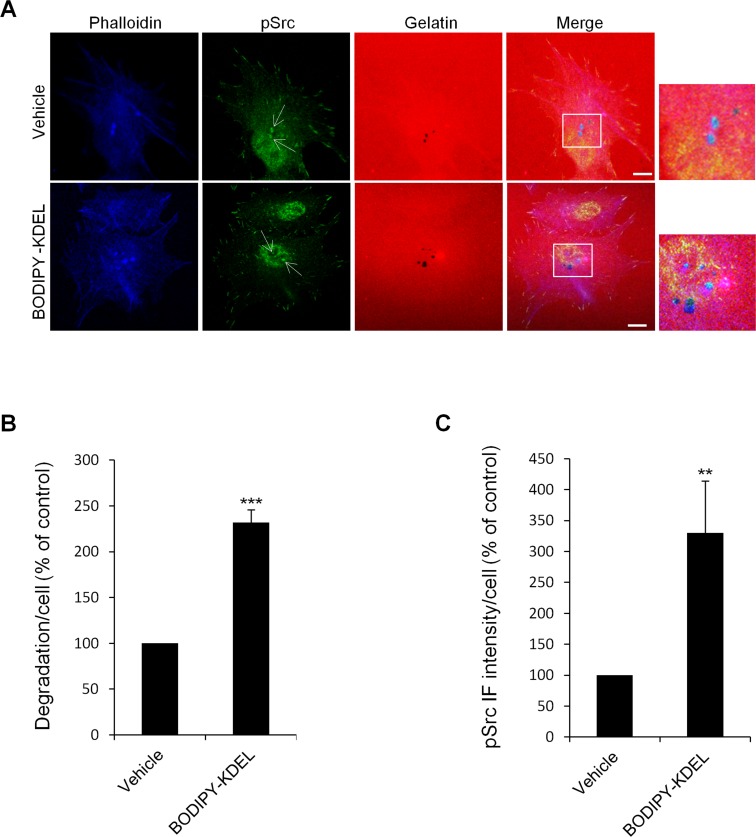
KDELR stimulation by Bodipy-KDEL activates Src to invadopodia (A) A375MM cells were grown on rhodamine-conjugated crosslinked gelatine (red) for 16 h in the presence of BB94. Following BB94 wash-out, the cells were incubated for a further 3 h with the membrane permeant KDELR agonist Bodipy-KDEL (3 μM) or with vehicle alone (Vehicle) as a control. After fixing, the cells were stained for pSrc (pTyr 419, green) and phalloidin (blue). Merged images of red, green and blue signals are also shown (Merge). pSrc immunofluorescence overlapping the invadopodia are shown in the enlargements of the boxed regions (small right panels: red green and blue signals). White arrows point to pSrc spots at invadopodia. Scale bars, 10 μm. The images are representative of two independent experiments. (B) Quantification of the degradation area per cell. Data are degradation area per cell (% of control), as means ±SEM of two independent experiments, with at least 50 cells quantified per experiment. *** p<0.001, compared to Vehicle cells (t-test). (C) Quantification of pSrc immunofluorescence at invadopodia. Data are means ±SEM of pSrc immunofluorescence per cell (% of control), from two independent experiments, with at least 50 cells quantified per experiment. ** p<0.001, compared to Vehicle cells (t-test).

We also activated the KDELR acutely by Bodipy-KDEL and measured the pSrc levels at the invadopodia. Bodipy-KDEL-treated cells were fixed and labelled with phalloidin for actin, and with an *anti*-*p*Src *antibody.* The degradation area of Bodipy-KDEL–treated cells was more than two times that of control cells (Fig. 7A, B). The pSrc levels at mature invadopodia were increased by four-fold in Bodipy-KDEL-treated cells, as compared to controls (Fig. 7A, C). Similar results were obtained when the analysis of the active Src was carried out in Bodipy-KDEL treated A375MM cells by Western blotting. The incubation with Bodipy-KDEL induced a marked progressive activation of Src as assessed by the pSrc bands shown in Suppl. Fig. 6.

Finally, we measured the levels of pSrc at the invadopodia of KDELR-depleted cells. Here, the pSrc levels decreased by 80% in the degradation areas of cells treated with siRNA for KDELR1 (Fig. 6E), and by 70% in the cells treated with siRNA for KDELR2 (Fig. 6F).

Collectively, these data indicate that KDELR1- and KDELR2-depletion regulate Src phosphorylation at the invadopodia, and suggest that this effect is responsible for the regulation of the ECM degradation process.

### KDELR activation promotes the phosphorylation of cortactin at the invadopodia

**Figure 8:**
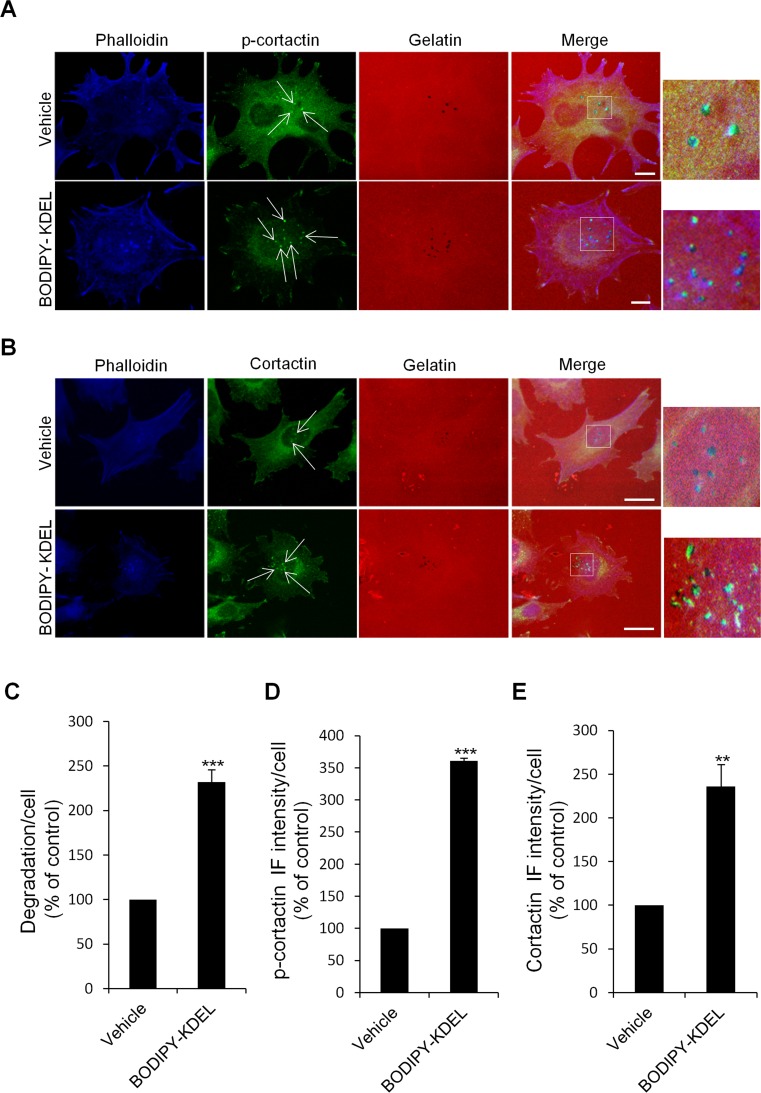
KDELR stimulation by Bodipy-KDEL triggers the phosphorylation of cortactin at invadopodia (A) A375MM cells were grown on rhodamine-conjugated crosslinked gelatine (red) for 16 h in the presence of BB94. Following BB94 wash-out, the cells were incubated for a further 3 h with the membrane permeant KDELR agonist Bodipy-KDEL (3 μM) or with vehicle alone (Vehicle) as a control. After fixing, the cells were stained for p-cortactin (Tyr 421 of cortactin, green) and phalloidin (blue). Merged images of red, green and blue signals are also shown (Merge). p-cortactin immunofluorescence overlapping the invadopodia are shown in the enlargements of the boxed regions (small right panels: red green and blue signals). White arrows point to p-cortactin spots at invadopodia. (B) KDELR stimulation increases cortactin to invadopodia. A375MM cells were treated as in A, fixed, and stained for cortactin (green) and phalloidin (blue). Merged images of red, green and blue signals are also shown (Merge). Cortactin immunofluorescence overlapping the invadopodia are shown in the enlargements of the boxed regions (small right panels: red green and blue signals). White arrows point to cortactin spots at invadopodia. (A, B) The images are representative of three independent experiments. Scale bars, 10 μm. (C) Quantification of the degradation area per cell. Data are degradation area per cell (% of control), as means ±SEM of three independent experiments, with at least 50 cells quantified per experiment. *** p<0.001, compared to vehicle cells (t-test). (D) Quantification of p-cortactin immunofluorescence at invadopodia. Data are means ±SEM of p-cortactin immunofluorescence per cell (% of control), from three independent experiments, with at least 50 cells quantified per experiment. *** p<0.001, compared to vehicle treated cells (t-test). (E) Quantification of cortactin immunofluorescence at invadopodia. Data are means ±SEM, as indicated for C. **p <0.01, compared to vehicle treated cells (t-test).

Src controls invadopodia formation/function by phosphorylating different substrates, including cortactin and ASAP1 [32, 34, 46]. Cortactin is a cytoskeletal protein enriched at invadopodia that is required for invadopodia formation and function [47]. Src dependent phosphorylation of cortactin promotes branched actin assembly by activating the ARP2/3 complex [46]. Prompted by the above results, which indicate an important role of the KDELR-Src signalling in the formation of invadopodia, we investigated the involvement of cortactin phosphorylation in this pathway. A375MM cells were placed on gelatine and treated for 3 h with Bodipy-KDEL as described above. The cells were then labelled with an antibody specific to the phosphorylated Tyr 421 of cortactin (p-cortactin) (Fig. 8A), a well known Src target of phosphorylation. The amount of p-cortactin at the invadopodia (phalloidin positive dots overlapping the degradation patches) increased markedly in Bodipy-KDEL-treated as compared to control cells (Fig. 8D).

We also analyzed cortactin phosphorylation in Bodipy-KDEL-treated A375MM cells by Western blotting. The incubation with Bodipy-KDEL induced a progressive phosphorylation of cortactin as assessed by the p-cortactin band shown in Suppl. Fig. 6. In addition, we observed an higher amount of cortactin at the invadopodia of cells treated with Bodipy-KDEL as compared to controls (Fig. 8B, E). These data support the idea that the KDELR controls the machinery of invadopodia formation/function.

### KDELR–Golgi–Src signalling controls ECM degradation via ASAP1 phosphorylation

ASAP1 is a phosphoinositide-dependent Arf-GAP multidomain protein, the depletion of which inhibits invadopodia formation, matrix degradation, and chemoinvasion [31, 48, 49]. Furthermore, ASAP1 expression in uveal melanoma, mammary carcinoma, and prostate cancer correlates with tumour invasiveness [31, 50, 51]. We thus examined the involvement of ASAP1 as downstream target of KDELR signalling.

**Figure 9:**
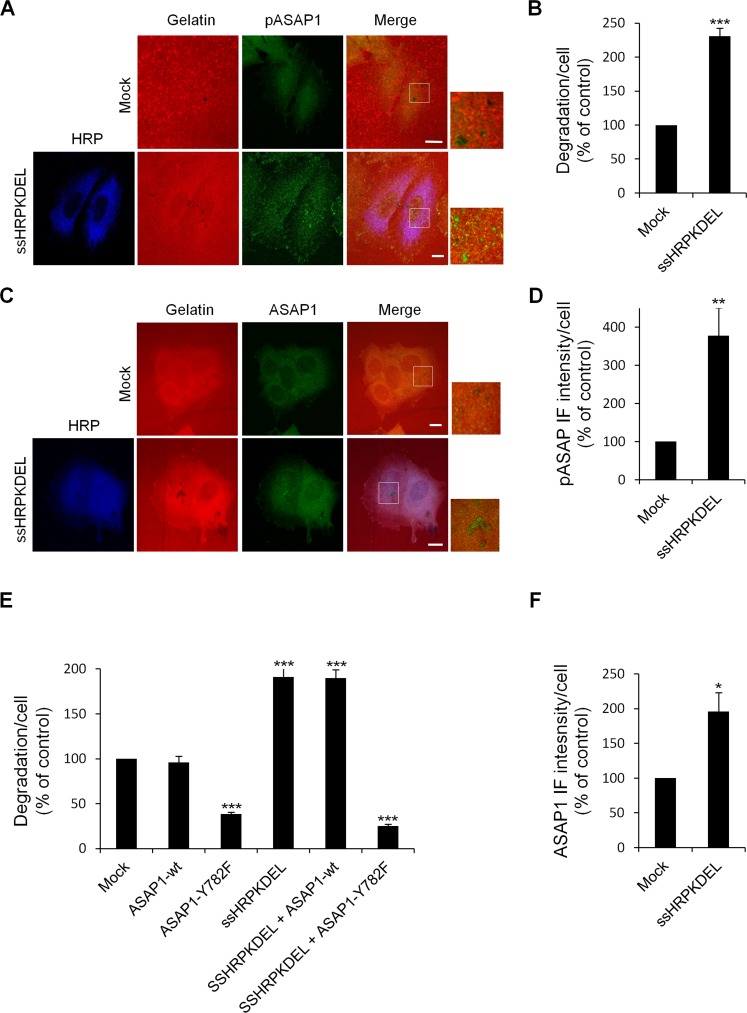
ASAP1 is involved in KDELR-dependent ECM degradation (A) KDELR stimulation increases the levels of phosphorylated ASAP1 in areas of ECM degradation. A375MM cells were transfected with the empty vector (Mock) or ssHRP^KDEL^, and grown on rhodamine-conjugated crosslinked gelatine (red) for 16 h in the presence of BB94. Following BB94 wash-out, the cells were incubated for a further 3 h and then fixed and stained for pASAP1 (pTyr 782, green). An anti-HRP antibody (blue) was used to visualise ssHRP^KDEL^-transfected cells. Merged images of red and green (Mock) and red, green and blue signals are also shown (Merge). pASAP1 immunofluorescence overlapping the degradation patches are shown in the enlargements of the boxed regions (small right panels: red and green signals). The images are representative of three independent experiments. (B) Quantification of the degradation area per cell. Data are degradation area per cell (% of control), as means ±SEM of three independent experiments. In each experiment, at least 100 cells were quantified. (C) KDELR stimulation increases ASAP1 recruitment to areas of degradation. A375MM cells were treated as in A, fixed, and stained for ASAP1 (green). An anti-HRP antibody (blue) was used to visualise ssHRP^KDEL^-transfected cells. Merged images of red and green (Mock) and red, green and blue signals are also shown (Merge). ASAP1 immunofluorescence overlapping the degradation patches are shown in the enlargements of the boxed regions (small right panels: red and green signals). The images are representative of three independent experiments. (D) Quantification of pASAP1 immunofluorescence in the cell regions overlapping the ECM degradation patches. Data are means ±SEM of pASAP1 immunofluorescence per cell (% of control), of three independent experiments. In each experiment at least 100 cells were quantified. (A, C) Scale bars, 10 μm. (E) The ASAP1 Y782F mutant inhibit ECM degradation. A375MM cells were transfected with empty vector (Mock), Flag-tagged ASAP1-wt or the Flag-tagged ASAP1 Y782F mutant. The cells were also transfected with myc-tagged ssHRP^KDEL^, myc-tagged ssHRP^KDEL^ and ASAP1-wt, or myc-tagged ssHRP^KDEL^ and ASAP1 Y782F. Following BB94wash-out, the cells were incubated for a further 3 h, then fixed, stained and scored for degradation of the ECM. Quantification of the degradation area per cell. Data are means ±SEM, as indicated for B. ***p<0.001, ASAP1 Y782F *versus* Mock; ssHRP^KDEL^ *versus* Mock; ssHRP^KDEL^ + ASAP1 Y782F *versus* ssHRP^KDEL^ (t test). (F) Quantification of ASAP1 immunofluorescence intensity in the cell regions overlapping the ECM degradation patches. Data are means ±SEM of ASAP1 immunofluorescence per cell (% of control), of three independent experiments. In each experiment at least 100 cells were quantified. (B, D, F) ***p <0.001, **p <0.01, *p <0.05 compared to Mock cells (t-test).

**Figure 10:**
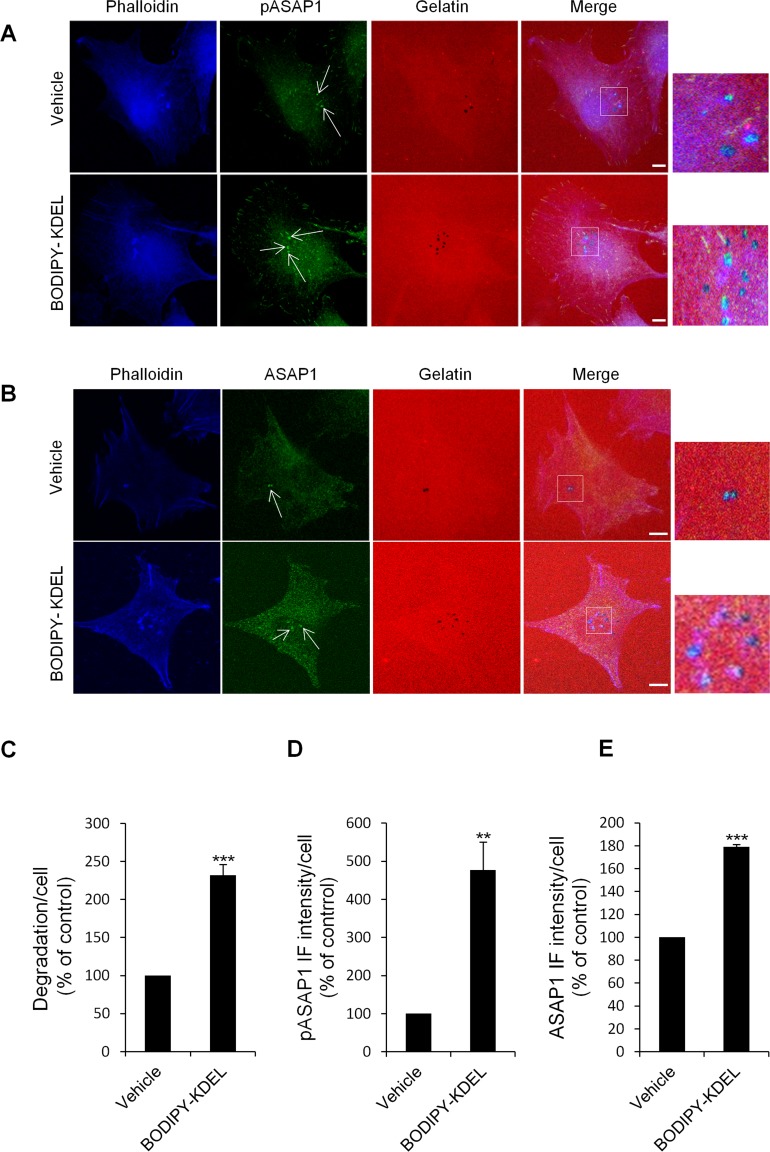
KDELR stimulation by Bodipy-KDEL triggers the phosphorylation of ASAP1 at invadopodia (A) A375MM cells were grown on rhodamine-conjugated crosslinked gelatine (red) for 16 h in the presence of BB94. Following BB94 wash-out, the cells were incubated for a further 3 h with the membrane permeant KDELR agonist Bodipy-KDEL (3 μM) or with vehicle alone (Vehicle) as a control. After fixing, the cells were stained for pASAP1 (pTyr 782, green) and phalloidin (blue). Merged images of red, green and blue signals are also shown (Merge). pASAP1 immunofluorescence overlapping the invadopodia are shown in the enlargements of the boxed regions (small right panels: red, green and blue signals).. White arrows point to pASAP1 spots at invadopodia. (B) KDELR stimulation increases ASAP1 to invadopodia. A375MM cells were treated as in A, fixed, and stained for ASAP1 (green) and phalloidin (blue). Merged images of red, green and blue signals are also shown (Merge). ASAP1 immunofluorescence overlapping the invadopodia are shown in the enlargements of the boxed regions (small right panels: red, green and blue signals). White arrows point to ASAP1 spots at invadopodia. (A, B) The images are representative of three independent experiments. Scale bars, 10 μm. (C) Quantification of the degradation area per cell. Data are degradation area per cell (% of control), as means ±SEM of three independent experiments, with at least 50 cells quantified per experiment. (D) Quantification of pASAP1 immunofluorescence levels at invadopodia. Data are means ±SEM of pASAP1 immunofluorescence per cell (% of control), from three independent experiments, with at least 50 cells quantified per experiment. (E) Quantification of ASAP1 immunofluorescence levels at invadopodia. Data are means ±SEM, as indicated for D. (C, D, E) ***p <0.001, **p <0.001 compared to vehicle treated cells (t-test).

First, we evaluated whether KDELR stimulation increases the levels of phosphorylated Tyr782 (a known Src substrate) of ASAP1 (pASAP1) in the cell regions overlapping the ECM degradation patches. To address this point, A375MM cells were transfected with ssHRP^KDEL^ and subjected to the ECM degradation assay, then fixed and stained with an *anti*-*p*ASAP1 *antibody*. As a control, the cells were transfected with the empty vector and processed in an identical fashion. The ssHRP^KDEL^-transfected cells showed greater ECM degradation area, as compared to the control cells (Fig. 9A, B), and the pASAP1 levels in the cell regions overlapping the degradation patches increased markedly (three-fold) (Fig. 9A, D). The pASAP1 levels at invadopodia were also evaluated in A375MM cells treated with Bodipy-KDEL, and labelled with phalloidin and pASAP1 (Fig. 10A). Besides the increase in ECM degradation, we observed a four-fold increase of pASAP1 levels also at mature invadopodia (Fig. 10A, C, D). We also analyzed ASAP1 phosphorylation in Bodipy-KDEL treated A375MM cells by Western blotting. The incubation with Bodipy-KDEL induced a progressive phosphorylation of ASAP1 as assessed by the pASAP1 band shown in Suppl. Fig. 6.

We then asked whether the amount of ASAP1 at the invadopodia contributes to the increase in pASAP1 detected upon KDELR stimulation. The cells were thus treated with Bodipy-KDEL, subjected to the ECM degradation assay, and then stained with an antibody against ASAP1 (Fig. 10B). The Bodipy-KDEL-treated cells almost doubled the amount of ASAP1 to invadopodia, as compared to the control cells (Fig. 10E). Similar results were obtained upon stimulating the KDELR by ssHRP^KDEL^ (Fig. 9C, F). These data indicate that KDELR activation causes an increase of ASAP1 levels to the invadopodia, and a Src-dependent phosphorylation of ASAP1.

Finally, we examined whether KDELR-dependent phosphorylation of ASAP1 is necessary to increase ECM degradation. To this end, we used a Tyr mutant of ASAP1, in which Tyr782 is replaced with the non-phosphorylatable phenylalanine (ASAP1-Y782F). It has been reported that this ASAP1-Y782F mutant inhibits podosome/invadopodia formation in MDA-MB-231 breast cancer cells [32], although its effect on ECM degradation is unknown. A375MM cells were thus transfected with the empty vector, ASAP1 wild-type (ASAP1-wt), or the ASAP1-Y782F mutant, separately or in combination with ssHRP^KDEL^, to evaluate the effects of the non-phosphorylatable mutant on basal and KDELR-dependent degradation. The ECM degradation in ssHRP^KDEL^-transfected cells almost double that of control cells, while the extent of degradation of ASAP1-wt–transfected cells was comparable to that in the controls (Fig. 9E). Similarly, the degradation areas of cells overexpressing both ASAP1-wt and ssHRP^KDEL^ were comparable to those of cells transfected with ssHRP^KDEL^ alone (Fig. 9E). Thus, ASAP1-wt does not influence basal or KDELR-stimulated ECM degradation. In contrast, the ASAP1-Y782F mutant inhibits both the ECM degradation of controls and ssHRP^KDEL^ stimulated cells (Fig. 9E).

These data indicate that the KDELR controls ECM degradation through Src-dependent regulation of ASAP1 phosphorylation.

## DISCUSSION

We have reported that transport from the ER to the Golgi complex during membrane trafficking activates the KDELR and a Src-dependent signal transduction cascade that is initiated through a pool of Gq located at the Golgi complex [6]. Here, prompted by the central role of Src in cell invasion, we have assessed whether the KDELR-Src pathway might control ECM degradation, a key process in cell invasion and growth [52, 53].

The results indicate that the activation of the KDELR1 and KDELR2 isoforms potently promotes the assembly of invadopodia and ECM degradation. These effects are associated with the activation of Src and the Src-dependent phosphorylation of cortactin at tyrosine 421 and of ASAP1 on tyrosine 782 at the invadopodia. Both the ASAP1 and cortactin phosphorylations are well known to be involved in ECM degradation; moreover, we directly show that the latter reaction is required to induce ECM degradation under our conditions. Given the established role of Src as a master controller of invadopodia formation and ECM degradation (active Src is both necessary and sufficient to induce the above events) [29, 30], it is logical to envisage a scheme in which the activation of the KDELR determines the activation of Src and the consequent phosphorylation of key Src substrates at sites where invadopodia are assembled and degradation activated.

ECM degradation is a key process in invasion and in the release of growth factors from the matrix [52, 53], hence in growth. Our findings point to the KDELR as a new major player in these cellular events. A comparison of the contribution of the KDELR to ECM degradation with that of serum, the most effective known degradation inducer, shows that the stimulated KDELR enhances ECM degradation to the extent obtained with high serum concentrations (30%-50%). Notably, the effects of these two stimuli (serum and the KDELR) are at least partially additive; thus, even when the cells are maximally stimulated by high serum, overexpression of the KDELR can further enhance ECM degradation. Possibly, a large pool of Src is available for the induction of invadopodia, and can be engaged either by growth factors or by KDELR to enhance cell degradation performance and invasivity.

The only known cellular ligands/activators of the KDELR are the ER chaperones that leave the ER and reach the Golgi with membrane traffic. Abundant literature data support the idea that chaperones are retrieved back from post ER compartments via the KDEL receptor [45, 54, 55]. Most chaperones leave the ER slowly because of retention mechanism, except a group of chaperones that includes ERp44 and ERP18, which leave the ER freely (apparently by bulk flow) and are kept in the ER solely by the KDELR retrieval system [44]. These might be the main endogenous activators of the KDELR. In any case, regardless of the identity of the chaperone acting as KDELR activatory ligands, the intensity of ER-to-Golgi traffic and the amount of suitable chaperones available for cycling between these two organelles are likely to increase the propensity of cells to degrade the matrix and invade, presumably in response to external directional cues. Are there physiological or pathological conditions under which the above mechanisms might play a functional role? An example might be tumour invasion, at least under certain specific circumstances. The solid tumour microenvironment is characterised by hypoxia, low glucose, and acidosis, all conditions that promote the unfolded protein response (UPR) and consequently the overexpression of chaperones. This might result in a higher flux of chaperones that leave the ER and activate the KDELR at the Golgi, which, in turn, might facilitate local invasive growth, metastatic dissemination and escape of the noxious habitat of the solid tumour, as has been reported to occur under the above mentioned conditions. In support of this hypothesis, Aoe a co-worker showed that UPR inducers such as thapsigargin increase the secretion of chaperones [56, 57], and that this treatment is sufficient to redistribute the KDELR from the Golgi to the ER [56]. Such a redistribution is considered the consequence of chaperone binding to the KDELR [58]. Thus, the KDELR-dependent matrix degradation might be related to the ability of cancer cells to tolerate and evade stressful situations typical of the cancer microenvironment.

In sum, events related to the cycling of the KDELR at the interface between the ER and the Golgi appear to be mechanistically interconnected with the ability of certain cells to invade the extracellular matrix and to grow. The full significance of this previous unsuspected link between these two different cellular activities remains to be explored under conditions of interest in specific cell types.

## MATERIALS AND METHODS

### Antibodies and constructs

The antibodies used were: anti-pSrc (p-Tyr 418; Invitrogen); anti-Src (SC18; Santa Cruz Biotechnology); anti-pASAP1 (p-Tyr782; Rockland Immunochemicals); anti-ASAP1 (Transduction Laboratories); anti-cortactin and anti p-cortactin (p-Tyr 421; Merck Millipore) anti-KDELR (Stressgen Biotechnology); anti PDI (Assay Designs); anti-KDELR 2, anti-KDELR 3, and anti-HRP antibodies (Abcam); anti-myc (Invitrogen), anti-actin (Sigma Aldrich); fluorophore-conjugated antibodies and fluorophore-conjugated actin (Molecular Probes); anti-HRP (Abcam) and HRP-conjugated antibodies (Calbiochem). The expression vectors used were: ssHRP and ssHRP^KDEL^ (D.F. Cutler, MRC, London, UK); KDELR-GFP isoforms 1, 2, 3a and KDELR-myc isoforms 1, 2, 3a were obtained by manipulating the original cDNA clones from I.M.A.G.E.; KDELR-D193N-GFP (V. Hsu, Harvard Medical School, Boston, MA, USA); KDELR-D193N-myc from subcloning the KDELR coding sequence from KDELR-D193N-GFP into a myc-containing modified pCMV5 vector; and ASAP1b-wt-Flag and ASAP1b-Y782F-Flag (P.A. Randazzo, National Cancer Institute, Bethesda, USA).

### Cell culture and cDNA transfection

The human melanoma A375MM and breast adenocarcinoma MDA-MB-231 cells were grown under standard conditions, as previously described [42]. The cells were plated at 50% confluence in 6-well plates and transfected using TransFast reagent (Promega, Madison, WI, USA) or Lipofectamine (Invitrogen), according to manufacturer instructions.

### Microscopy

Immunofluorescence microscopy was as described previously [6]. Confocal images were acquired using a Zeiss LSM510 inverted confocal microscope system (Carl Zeiss, Gottingen, Germany). Fixed cells were analysed using a 63× oil-immersion objective, maintaining the pinhole of the objective at 1 Airy unit. Images were acquired under non-saturating conditions (pixel fluorescence below 255 arbitrary units) and using the same settings for all samples.

### Fluorophore-conjugated gelatine preparation, gelatine-coated coverslip preparation, and ECM degradation assay

Preparation of rhodamine B (Sigma-Aldrich) conjugated porcine gelatine (Sigma-Aldrich) was performed according to the method of Mueller and Chen [59], with the modifications reported in [60, 61]. Fluorescent gelatine-coated coverslips were prepared and the assay carried out as described previously [41, 42]. The ECM degradation assay was performed according to the previously published invadopodia synchronisation protocol with some modifications [47]. Briefly, the cells were plated on gelatine-coated coverslips in medium containing 5 μM BB94, a broad-range matrix metalloprotease inhibitor (British Biotech, UK). After 16 h, BB94 was washed-out to allow synchronous invadopodia formation, and the cells were fixed at 3 h and processed for immunofluorescence. The cells to be transfected were plated at 30% confluence in six-well plates. The following day, they were transfected as described above. Then, 6 h after transfection, the cells were detached, plated on gelatine-coated coverslips for 16 h, and processed as described above.

### Quantification of degradation areas and immunofluorescence signals in invadopodia

Areas of degradation were defined as dark patches in the fluorophore-conjugated gelatine matrix underlying the cells. In all, 50 or 100 cells (from two wells) per point per experiment were acquired, as reported above and in [42]. The total area of degradation patches was automatically determined using the histogram function of LSM510-3.2 software (Zeiss). The total degradation area for each condition was then normalised for cell number. The total immunofluorescence intensity of the signals to be quantified was measured at the cell regions overlapping the ECM degradation patches or at invadopodia (phalloidin dots overlapping the degradation patches) as indicated. The total immunofluorescence intensity for each condition was then normalised for cell number. All experiments were carried out at least two times. The results are shown as arbitrary units (AU).

### RNA interference

The cells were transfected with 100 nM of the siGENOME™ SMARTpool® reagents (Dharmacon, Lafayette, CA, USA) containing four pooled siRNA duplexes against human KDELR1, KDELR2 and KDELR3 using Lipofectamine 2000 (Invitrogen, CA, USA) according to the manufacturer instructions. Two independent siRNAs duplexes for KDELR1 and KDELR2 were also used, mamely KDELR1-A: GUUCAAAGCUACUUACGAU, KDELR1-B: GGUGUUCACUGCCCGAUAU; KDELR2-A: ACACAUCUAUGAAGGUUAU; KDELR2-B: CCUUCAGGGUGCUCGGACA (IDT Integrated DNA Technologies). The cells were plated on gelatine-coated coverslips 72 h after siRNA treatment in the presence of 5 μM BB94, and incubated at 37 °C in the presence of 5% CO_2_ for a further 24 h. The ECM degradation and invadopodia formation were evaluated as described above.

### Immunoblot analysis

The silencing efficiency was evaluated by Western blotting 72-96 h after transfection. Briefly, cells were lysed in 1% Triton-X100, 1 mM EDTA, 1 mM β-mercaptoethanol, 1 mM iodoacetamide, plus protease and phosphatase inhibitors (Roche). The lysates were passed through a syringe (15 times), incubated with rotation for 1.5 h at 4 °C, cleared by centrifugation (20,000× *g*, 15 min, 4 °C), then added to 2× SDS sample buffer and incubated at room temperature for 15 min prior to electrophoresis. Cell lysates were separated on 12% SDS-polyacrylamide gels and subjected to Western blotting. ssHRP-KDEL expression levels were evaluated 24 hours post transfection using the same protocol except for the absence of iodacetamide. The phosphorylation levels of Src, ASAP1 and cortactin were evaluated as above except for the lysis buffer that was replaced with the RIPA buffer (150 mM NaCl, 20mM Tris pH 8.0, 0.1% SDS, 0.5% sodium deoxycholate, 1% Triton-X 100). Further, the cell lysates were cleared by centrifugation.

### Quantitative real time PCR

Total RNA was extracted by trizol reagent (Invitrogen). One μg of RNA was reverse transcribed using the superscript kit (Invitrogen) according to the manufacturer's instructions. Four and 10 ng of cDNA was amplified by Real Time PCR System from Applied Biosystems. Primer sequences: KDELR1 forward primer 5′ ATT CTG GCG TTC CTG GTC AAT 3′; KDELR1 reverse primer 5′ TTG AAG AGA TAG AGC GTG CGG 3′; KDELR3 forward primer 5′ TCC GCC TGG AGT TTC TTC TG 3′; KDELR3 reverse primer 5′ AAG AGC TGG GGC AGG ATA GC 3′; KDELR2 primers were from Qiagen, code number QT02399324. Data were calculated as fold change compared to controls using the 2^(−Delta Delta Ct)^ method, using actin as a housekeeping control gene for normalization. All reactions were performed in duplicate.

### Preparation of membrane-permeant peptides and treatment of cells subjected to the ECM degradation assay

The conjugation of the CFFKDEL and CFFKDEA peptides (Gen Script, USA) with Bodipy was performed as previously described [62]. For treatment, the cells were grown on gelatine-coated coverslips for 16 h in the presence of 5 μM BB94 in complete medium at 30% to 50% confluence. The BB94 was washed-out and cells were incubated in Opti-MEM containing Bodipy-KDEA or Bodipy-KDEL (3 μM) for 3 h. The cells were then processed for immunofluorescence and ECM degradation, quantified as described above.

## SUPPLEMENTARY MATERIAL FIGURES


