# Supplementary material for: A Golgi-based KDELR-dependent signalling pathway controls extracellular matrix degradation
Source: Oncotarget. 2014 Dec 18;6(5):3375–93. doi: 10.18632/oncotarget.3270 (PMC4413660; doi:10.18632/oncotarget.3270)
Supplement: Supplementary file 1 [file oncotarget-06-3375-s001.pdf]

A Golgi-based KDELR-dependent signalling pathway controls extracellular matrix degradation

Supplementary Material

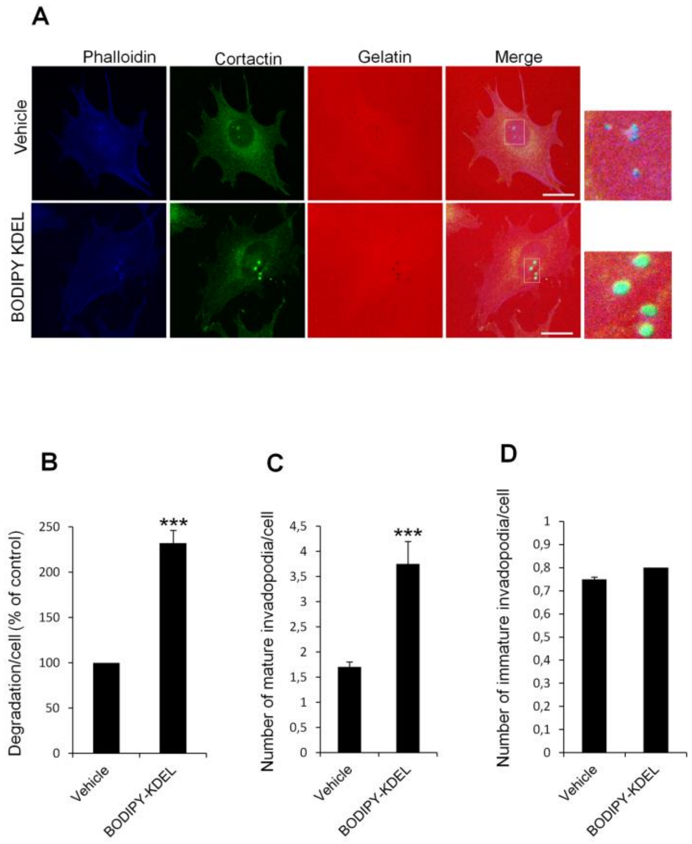

Supplementary Figure 1: KDELR stimulation by Bodipy-KDEL promotes ECM degradation and invadopodia formation.

(A) A375MM cells were grown on rhodamine-conjugated crosslinked gelatine (red) for 16 h in the presence of BB94. Following BB94 wash-out, the cells were incubated for a further 3 h with the membrane permeant KDELR agonist Bodipy-KDEL (3 µM) or the vehicle (as indicated). After fixing, the cells were stained with phalloidin (blue) and cortactin (green). Merged images red,green and blue signals are shown (Merge). Invadopodia are shown in the enlargements of the boxed regions (small right panels: green and red signals). Scale bars, 10 µm. The images are representative of two independent experiments. **(B)** Quantification of the degradation area per cell is expressed as % of control. **(C)** Quantification of the number of mature invadopodia per cell. **(D)** Quantification of the number of immature invadopodia per cell. Data are means ±SEM of two independent experiments, with at least 50 cells quantified per experiment. *** $p<0.001$, compared to vehicle treated cells (t-test).

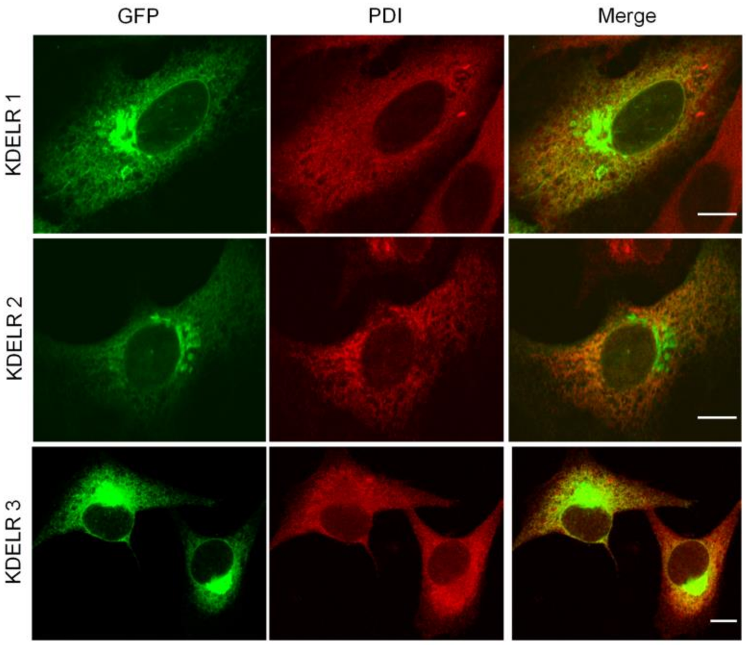

Supplementary Figure 2: High expression levels of KDELRs induce the redistribution of the KDELR itself to the ER.

A375MM cells transfected with KDELR1-GFP, KDELR2-GFP or KDELR3-GFP, were fixed 24 h later and stained with anti-PDI (red). Cells expressing high levels of KDELR1-GFP, KDELR2-GFP or KDELR3-GFP are shown. Scale bars, 10 μm. The images are representative of at least three independent experiments.

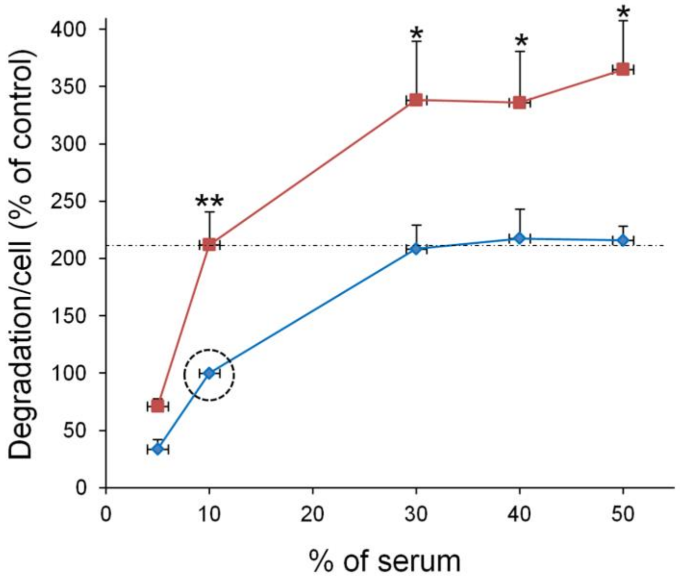

Supplementary Figure 3: KDELR2-overexpressing cells exposed to different serum concentrations stimulate ECM degradation at all serum concentration.

A375MM cells were transfected with empty vector (Mock) or KDELR2-myc and grown on rhodamine-conjugated cross-linked gelatine for 16 h in the presence of BB94. Following BB94 wash-out, the cells were incubated for a further 3 h in the presence of different serum concentrations, from 0% to 50%. Data are means ±SEM of degradation area per cell expressed as % of control: mock transfected, 10% of serum (circled data point), from three independent experiments, with at least 100 cells quantified per experiment. **p<0.01, *p<0.05 compared to mock transfected cells, 10% of serum (t-test). Dashed line indicates that the transfection of KDELR2 as well as high serum lead to an analogous levels of ECM degradation stimulation in comparison with standard conditions (mock transfected cells, 10% of serum).

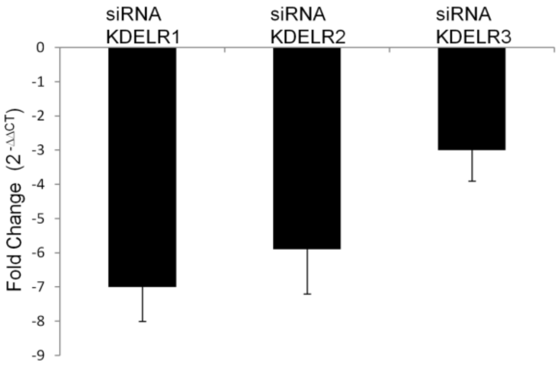

Supplementary Figure 4: Efficiency of KDELR knockdown by real time PCR.

A375MM cells were treated without (mock) or with siRNAs targeting KDELR1, KDELR2 and KDELR3 (siRNA KDELR) for 96 h. KDELR1, KDELR2, and KDELR3 expression levels were assessed by qPCR using receptor specific primers. Expression levels were normalized with those obtained from the amplification of actin and expressed as fold change compared to mock. Folds are means ±SEM from three independent experiments.

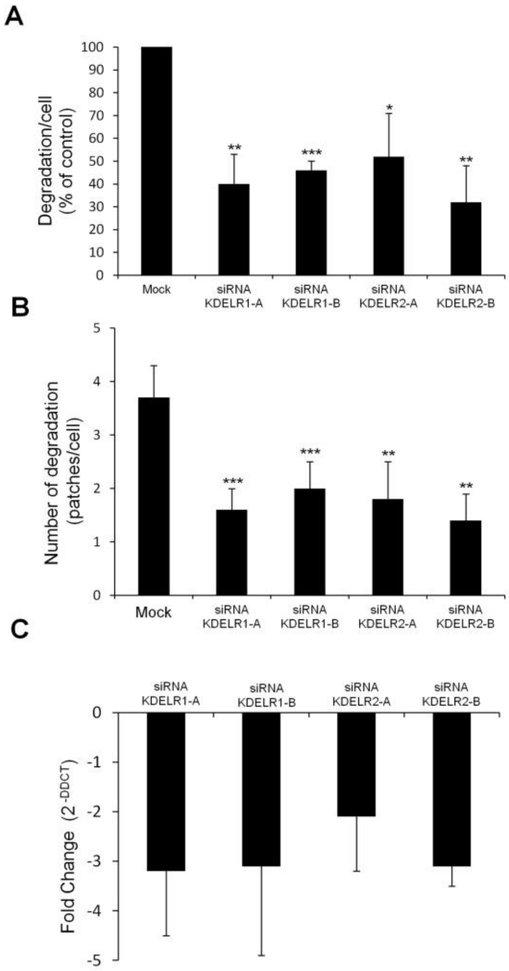

Supplementary Figure 5: Independent KDELR siRNAs confirm the involvement of this receptor in ECM degradation.

(A) A375MM cells were treated without (mock) or with siRNAs targeting KDELR1 and KDELR2. Two siRNAs for KDELR1 (siRNA KDELR1-A and siRNA KDELR1-B) and two siRNAs for KDELR2 (siRN KDELR2-A and siRNA KDELR2-B) have been used independently.

Seventy two hours post interference cells were plated for 24 h on rhodamine-conjugated gelatine in the presence of BB94. Following the BB94 wash-out, the cells were incubated for a further 3 h, then fixed and scored for their ability to degrade the ECM. Data are degradation area per cell (% of control), as means ±SEM from three independent experiments, with at least 50 cells quantified per experiment.. **(B)** Quantification of the number of degradation patches per cell in the experiment described in A. **(A, B)** ***p <0.001, **p <0.01, *p <0.05 compared to mock cells (t-test)**. (C)** A375MM cells were treated as in A, and then 96-h post interference KDELR1 and KDELR2 expression levels were assessed by quantitative real time PCR (qPCR) using receptor specific primers. Expression levels were normalized with those obtained from the amplification of actin and expressed as fold change compared to mock.

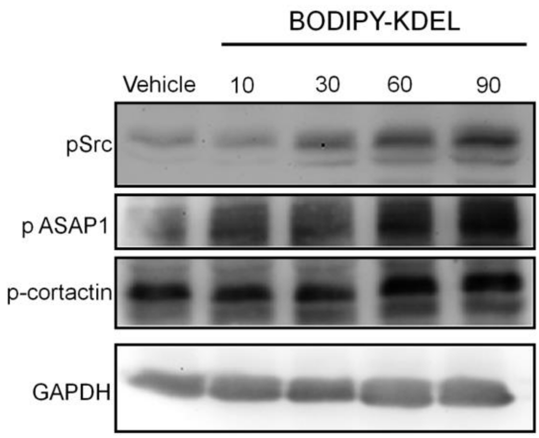

Supplementary Figure 6: KDELR stimulation induces the activation of Src and the phosphorylation of ASAP1 and cortactin.

(A) A375MM cells were incubated for 10 to 90 minutes, as indicated, with the membrane permeant KDELR agonist Bodipy-KDEL (3 μM) or with vehicle alone (Vehicle) as a control. Cells were lysed and analyzed by immunoblotting using multiple antibodies as indicated. GAPDH immunoblotting have been used as loading control.
